# Thrombin generation as a predictor of outcomes in patients with non-traumatic intracerebral hemorrhage

**DOI:** 10.3389/fneur.2022.912664

**Published:** 2022-08-18

**Authors:** Linda Lóczi, Rita Orbán-Kálmándi, Tamás Árokszállási, István Fekete, Klára Fekete, Máté Héja, Judit Tóth, László Csiba, Zsuzsa Bagoly

**Affiliations:** ^1^Division of Clinical Laboratory Sciences, Department of Laboratory Medicine, Faculty of Medicine, Kálmán Laki Doctoral School, University of Debrecen, Debrecen, Hungary; ^2^Department of Neurology, Faculty of Medicine, University of Debrecen, Debrecen, Hungary; ^3^Department of Radiology, Faculty of Medicine, University of Debrecen, Debrecen, Hungary; ^4^ELKH-DE Cerebrovascular Research Group, University of Debrecen, Debrecen, Hungary

**Keywords:** hemorrhagic stroke, thrombin generation, outcome, intracerebral hemorrhage, mortality

## Abstract

**Background:**

Non-traumatic intracerebral hemorrhage (ICH) accounts for 10–15% of all strokes and leads to a higher rate of mortality as compared to ischemic strokes. We aimed to find out whether the thrombin generation assay (TGA) could predict outcomes in patients with ICH.

**Patients and methods:**

In this prospective, observational study, 87 consecutive patients with ICH and 164 healthy controls were included. Computed tomography (CT), detailed clinical investigation, and laboratory investigations were performed from patients on admission. TGA was performed using stored platelet poor plasma obtained on admission. Lag time, endogen thrombin potential (ETP), peak thrombin, and time to peak parameters were calculated. Short- and long-term outcomes of ICH were defined at 14 days and 3 months post-event according to the NIHSS and the modified Rankin Scale (mRS), respectively.

**Results:**

Peak thrombin was significantly higher in patients as compared to controls (397.2 ± 93.9 vs. 306 ± 85.3 nM, *p* < 0.0001). Lag time, ETP, and time to peak parameters showed a significant positive correlation with CRP in both groups. In patients with worse long-term functional outcomes, peak thrombin was significantly higher as compared to those with favorable outcomes [mRS 2–6 median: 402.5 (IQR:344.8–473.8) vs. mRS 0–1: 326.4 (294.2–416.1) nM, *p* = 0.0096]. Based on the statistically optimal threshold of 339.1 nM peak thrombin, the sensitivity and specificity of this parameter to determine mRS 2–6 as an outcome were 80.8 and 64.7%, respectively. In a binary logistic regression model including age, sex, BMI, smoking status, NIHSS on admission, D-dimer, and peak thrombin (>339.1 nM), only NIHSS and the peak thrombin parameters remained in the model as significant, independent predictors of poor outcome. Lag time and time to peak showed a modest, significant negative correlation with intracerebral bleeding volume on admission (*r* = −0.2603, *p* = 0.0231 and *r* = −0.3698, *p* = 0.0010, respectively). During the follow-up of patients, estimated hemorrhage volumes on day 90 showed significant positive association with the ETP and peak thrombin parameters (*r* = 0.3838, *p* = 0.0363 and *r* = 0.5383, *p* = 0.0021, respectively).

**Conclusion:**

In patients with ICH, TG was increased as compared to healthy controls, which might be explained by the presence of higher inflammatory parameters in patients. Peak thrombin measured on admission might be a useful tool to predict outcomes in patients with ICH.

## Introduction

Stroke is a leading cause of death and disability in all developed countries. Non-traumatic intracerebral hemorrhage (ICH) constitutes ~10–15% of acute strokes and has the highest case fatality among all strokes, with ~40% of patients dying within the first month (1, 2). Despite the significant advances in acute ischemic stroke care, pharmacological treatment in ICH is still limited, and therefore, it remains the most devastating cerebral vascular disease (1). This is at least partly due to an existing knowledge gap in the pathophysiology of intracerebral bleeding and the mechanisms driving hematoma progression. Coagulation disorders and impaired hemostasis have been shown to increase the risk of ICH (3, 4). However, studies on associations between hemostasis abnormalities and the outcome of ICH remain limited. In a prospective series of patients with ICH, significant hemorrhage growth occurred in 38% of patients, which was associated with outcomes (5). Besides a handful of studies indicating that increased admission D-dimer levels predict mortality (6–8), the impact of the hemostasis system on the evolution of hematoma and the outcome of acute non-traumatic ICH has not been fully investigated.

Thrombin generation assay (TGA) is a global hemostasis test providing information about the speed and amount of generated thrombin in plasma (9). The analysis of the thrombin generation curve (thrombogram) reveals the concerted action of plasmatic pro- and anticoagulant factors; therefore, the assay has been suggested to be a promising candidate to demonstrate hypo-or hypercoagulability in patients. TGA has been shown to correlate with the clinical phenotype in hemophilia and with the response to treatment (10–12). TGA seems also potentially useful in the field of thrombotic disorders, to predict the risk of recurrent venous thromboembolism and to help tailor antithrombotic treatments (10, 12). The aim of this study was to evaluate whether the TGA correlates with the evolution of hematoma in patients with non-traumatic, spontaneous ICH and to see whether this test might serve as a useful tool to predict outcomes.

## Materials and methods

### Patients and controls

In this prospective observational study, consecutive patients with non-traumatic intracerebral hemorrhagic stroke (ICH) were enrolled in a single stroke center (Department of Neurology, University of Debrecen, Hungary). The study protocol was published earlier (13). Patient enrollment was initiated in June 2017 and was completed in September 2020. Inclusion criteria were the following: patients over 18 years of age with acute non-traumatic intracerebral hemorrhage, verified with non-contrast computerized tomography (NCCT) scan. Exclusion criteria included the presence of cerebral aneurysm, arteriovenous malformation, epidural hemorrhage, subdural hemorrhage, malignancy, severe hepatic and renal insufficiency, hemorrhagic diathesis, anticoagulant treatment, and SARS-CoV-2 infection at hospital admission or during follow-up. The presence of ICH was diagnosed by complex neurological examination based on the clinical symptoms, brain imaging using NCCT scan (if needed CT angiography and MRI were also performed). Follow-up NCCT scans were performed 14 days and 3 months after the event. CT images were analyzed simultaneously by 3 independent investigators and a comprehensive list of radiographic features and estimated ICH volume was recorded (14). A detailed list of demographic and clinical characteristics (age, sex, BMI, previous medications, history of cerebrovascular and cardiovascular diseases, cerebrovascular risk factors including smoking, time of symptom onset, etc.) was registered for each patient on admission. Stroke severity was determined by the National Institutes of Health Stroke Scale (NIHSS) on admission and on day 7 (15). Risk stratification of each patient was performed using the ICH score (based on GCS score, age, infratentorial origin, intraventricular hemorrhage, and ICH volume) (16). Patients were followed and long-term functional outcomes were determined at 3 months after the stroke event using the modified Rankin Scale (mRS) (17). As from March 2020, all patients were investigated about the potential acquisition and symptoms of SARS-CoV-2 infection on admission and during follow-up. In case of a suspected infection, patients underwent reverse transcriptase polymerase chain reaction testing of SARS-CoV-2 RNA extracted from nasopharyngeal/oropharyngeal swabs.

The following outcomes were investigated: (1) long-term outcome at 90 days post-event: mRS 0–1 was defined as favorable long-term outcome (18) (2) mortality by days 14 and 90.

As reference intervals for TGA parameters have not been published by the manufacturer, the results of patients were compared to that of healthy individuals. Inclusion criteria of healthy controls were as follows: over 18 years of age, willing to participate in the study, and signing informed consent. Exclusion criteria were as follows: chronic disorders (malignancy, known hemorrhagic or thrombotic disorder, autoimmune disorders, chronic kidney, or liver disease, etc.) except for moderate hypertension, acute illness within the past 4 weeks, thrombotic event, surgery, or major trauma within the past 3 months, and anticoagulant use.

### Informed consent

The study design was in accordance with the principles of the Declaration of Helsinki and was approved by the Institutional Ethics Committee of the University of Debrecen and the Ethics Committee of the National Medical Research Council. All patients or their relatives and all controls provided written informed consent.

### Blood sampling and laboratory measurements

Peripheral venous blood samples were taken from all patients on admission. Healthy control individuals were asked to provide blood samples in an outpatient setting. Routine laboratory tests (ions, glucose level, renal and liver function tests, high-sensitivity C-reactive protein measurement, and complete blood count) were carried out immediately by standard laboratory methods (Roche Diagnostics, Mannheim, Germany and Sysmex Europe GmbH, Hamburg, Germany). For the examination of hemostasis tests, blood samples were collected to vacutainer tubes containing 0.109 M sodium citrate (Becton Dickinson, Franklin Lane, NJ) and were processed immediately (centrifugation two times at 1,500 g, room temperature, 15 min). Screening tests of coagulation (prothrombin time, activated partial thromboplastin time, and thrombin time) were performed immediately on a BCS coagulometer using routine methods (Siemens Healthcare Diagnostic Products, Marburg, Germany). For the execution of thrombin generation assays and other specific hemostasis tests, aliquots of citrated plasma were labeled with a unique code and stored at −80°C until analysis. Specific hemostasis tests including thrombin generation were performed from stored plasma aliquots by the investigators who were blinded to patient identification and clinical data. Fibrinogen levels according to the method of Clauss, factor VIII (FVIII) activity using a chromogenic assay, and D-dimer levels using an immunoturbidimetric assay were measured on a BCS coagulometer by standard methods (Siemens Healthcare Diagnostic Products, Marburg, Germany).

### Thrombin generation measurements

The thrombin generation test was carried out as described previously using the Thrombinoscope CAT (Calibrated Automated Thrombogram, Maastricht, The Netherlands) assay according to the manufacturer's instructions (Diagnostica Stago, Asnières, France) (19, 20). Briefly, 20 μl PPP-Reagent^TM^ (containing 5 pM recombinant tissue factor and 4 μM phospholipids) was incubated with 80 μl of plasma for 10 min in black round-bottomed 96-well-microtiter plates. For each sample, 20 μl of calibrator (Thrombin Calibrator^TM^) was run in parallel to correct the fluorescence signal for substrate consumption and plasma color variability. The process of thrombin generation was initiated by the automatic dispense of 20 μl FluCa-Kit^TM^ (a mixture of fluorogenic substrate and Fluo-Buffer containing CaCl_2_) into each well. All samples were run in duplicates. Fluorescence was detected by a Fluoroskan Ascent^®^ fluorimeter (Thermo Fischer Scientific, Waltham, MA) and the generated curves were analyzed by the Thrombinoscope software (Thrombinoscope BV, Maastricht, The Netherlands). Thrombin generation curves were characterized by the following parameters (calculated and presented by the Thrombinoscope software): (1) lag time: defined as the moment when the signal deviates by more than 2 standard deviations from the baseline, (2) endogenous thrombin potential (ETP): the area under the curve, (3) peak thrombin: the highest thrombin concentration, (4) time to peak: the time to peak thrombin generation.

### Statistical analysis

Statistical analysis was performed using the Statistical Package for Social Sciences (SPSS, version 26.0, Chicago, IL) and GraphPad Prism 8.0 (GraphPad Prism Inc., La Jolla, CA). Normality of data was studied using the Shapiro–Wilk test. Student's *t*-test or the Mann–Whitney U test was performed for independent two-group analyses. Spearman's or Pearson's correlation coefficient was used to determine the strength of correlation between continuous variables. Differences between categorical variables were assessed by the χ^2^ test or by Fisher's exact where appropriate. Receiver operating characteristic (ROC) curve was built by plotting sensitivity vs. 1-specificity. Optimal threshold of a given test parameter was calculated based on Youden's J statistics. Assay characteristics of sensitivity, specificity, positive predictive value (PPV), and negative predictive value (NPV) were calculated using contingency table and the χ^2^ test based on the statistically optimal threshold value. A binary backward logistic regression model was used to determine independent predictors of long-term functional outcome. Adjustments of the models were based on the results of univariate analyses of baseline characteristics between groups, including the variables of age and sex. The results of the logistic regression analysis were expressed as odds ratio (OR) and 95% confidence interval (CI). A *p* < 0.05 was considered statistically significant.

## Results

In this study, 87 patients with non-traumatic, spontaneous ICH and 164 healthy controls were enrolled. Two patients with COVID-19 were not included in the study: in case of one patient SARS-CoV-2 infection was detected on admission, another patient acquired SARS-CoV-2 infection on day 25 after the event, and thus, this patient was also excluded from the study. The assumed cause of ICH was hypertension in all patients, as based on the exclusion criteria, other major causes of ICH, including cerebral aneurysm, arteriovenous malformation, malignancy, severe liver insufficiency, hemorrhagic diathesis, amyloidosis, or vasculitis were excluded. Baseline characteristics of the study population are shown in Table 1. The mean age of patients with ICH was 67 (± 12) years, and 63% of patients were men. Mean age of healthy controls was 53 (± 9) years, and 34% were men. The most frequent cerebrovascular risk factor was hypertension (97%) in patients. CRP and fibrinogen levels, WBC, and platelet count were significantly higher in patients with ICH as compared to controls. PT and APTT were significantly shorter in patients, whereas TT was marginally, but significantly longer in patients as compared to controls. D-dimer levels and FVIII activity were significantly higher in patients as compared to healthy controls. Stroke characteristics and outcomes of enrolled patients are shown in Table 2. Median NIHSS on admission was 13 (IQR: 6–19). Mortality was 23% by day 14 after the event and 41% by day 90. In terms of long-term functional outcome, only 20% of the patients had a favorable outcome (mRS 0–1) by the end of the 3rd month. Median volume of hemorrhage was 12.0 (IQR: 3.5–29.8) cm^3^ on admission. The median ICH score was 0 (IQR: 0–2). After the event of stroke, most patients received antihypertensive (89%) and antacid therapy (94%). Low molecular weight heparin (LMWH) prophylaxis was administered in case of 26 high-risk patients (30%); all of these patients had high degree of disability or died (mRS 2–6) by the end of the 3rd month. Antiplatelet drugs were not used during the follow-up. Four cases of thrombotic events (1 patient with deep vein thrombosis and 3 patients with pulmonary embolism) were registered during the follow-up.

**Table 1:** Baseline characteristics of the study population.

**Variables**	**Patients**	**Controls**	***p***
Number of individuals, *n*	87	164	-
Age, y, mean ± SD	67 ± 12	53 ± 9	<0.0001
Male sex, *n* (%)	55 (63)	55 (34)	<0.0001
Cerebrovascular risk factors, *n* (%)
Arterial hypertension	84 (97)	56 (34)	<0.0001
Hyperlipidemia	50 (57)	0	-
Diabetes mellitus	31 (36)	0	-
Active smoker	17 (20)	34 (21)	0.9646
BMI, kg/m^2^, median (IQR)	26.7 (23.8–31.2)	25.4 (23.4–28.0)	0.0695
Laboratory parameters on admission, median (IQR), or mean ± SD
Serum glucose, mmol/L	7.3 (5.8–9.8)	4.9 (4.6–6.2)	<0.0001
Triglyceride, mmol/L	1.5 (1.0–2.1)	1.4 (0.9–2.1)	0.3964
Total cholesterol, mmol/L	4.8 (4.3–5.7)	5.1 (4.4–5.8)	0.0776
HDL, mmol/L	1.5 (1.1–1.8)	1.4 (1.2–1.7)	0.4816
LDL, mmol/L	2.9 ± 1.0	3.3 ± 0.9	0.0233
Creatinine, μmol/L	69 (60–82)	67 (58–77)	0.3532
ASAT, U/L	23 (17–33)	19 (16–25)	0.0052
ALT, U/L	17 (13–24)	20 (15–26)	0.0450
LD, U/L	224 (199–254)	251 (183–350)	0.0747
hsCRP, mg/L	2.5 (1.1–6.2)	1.8 (0.8–3.4)	0.0031
WBC, G/L	8.52 (6.73–11.42)	6.95 (5.85–8.08)	<0.0001
RBC, T/L	4.57 (4.22–4.97)	4.67 (4.35–5.05)	0.2679
Hemoglobin, g/L	142 ± 16	140 ± 14	0.4432
Platelet count, G/L	226 (180–272)	249 (215–298)	0.0039
PT, s	8.1 (7.8–8.6)	9.2 (8.3–9.9)	<0.0001
INR	0.96 (0.93–1.01)	0.98 (0.68–1.01)	0.0214
APTT, s	26.7 (24.6–29.2)	28.4 (28.0–28.6)	0.0002
TT, s	16.6 (16.0–17.6)	16.3 (15.4–17.2)	0.0141
Fibrinogen, g/L	3.8 (3.1–4.5)	2.4 (1.9–3.2)	<0.0001
D-dimer, mg/L	0.63 (0.38–1.0)	0.28 (0.18–0.38)	<0.0001
FVIII activity, %	228 (153–289)	97 (83–123)	<0.0001

Continuous variables are expressed as mean ± SD or median (interquartile range). Categorical variables are indicated as number (percentage). ALT, alanine aminotransferase; APTT, activated partial thromboplastin time; ASAT, aspartate aminotransferase; BMI, body mass index; FVIII, coagulation factor VIII; HDL, high-density lipoprotein; hsCRP, high-sensitivity C-reactive protein measurement; INR, international normalized ratio; IQR, interquartile range; LD, lactate dehydrogenase; LDL, low-density lipoprotein; n, number; PT, prothrombin time; RBC, red blood cell count; SD, standard deviation; TT, thrombin time; WBC, white blood cell count.

**Table 2:** Stroke characteristics and outcomes in the investigated cohort.

**Variables**	
Stroke severity on admission, NIHSS, median (IQR)	13 (6–19)
Glasgow coma scale, median (IQR)	12 (9–14)
ICH score, median (IQR)	0 (0–2)
Imaging data, *n* (%)	
Presence of hydrocephalus on admission	
None	54 (62)
External hydrocephalus	3 (3)
Internal hydrocephalus	20 (23)
Both	10 (12)
Hemisphere localization of ICH on admission	
Left hemisphere	52 (60)
Right hemisphere	33 (38)
Bilateral hemisphere	2 (2)
Presence of intraventricular hemorrhage on admission	
None	52 (60)
Lateral ventricle	7 (8)
III. ventricle	1 (1)
IV. ventricle	1 (1)
Combined	26 (30)
Infratentorial origin	
Yes	5 (6)
No	82 (94)
Estimated volume of hemorrhage, cm^3^, median (IQR)	
On admission	12.0 (3.5–29.8)
Day 14	7.9 (2.5–24.0)
Day 90	0 (0.0–1.7)
Mortality by day 14, *n* (%)	20 (23)
Long-term outcome, mRS, day 90, *n* (%)	
mRS 0–1	17 (20)
mRS 2–5	33 (38)
mRS 6 (death)	36 (41)
Undetermined	1 (1)
Medication after the stroke event, *n* (%)	
LMWH prophylaxis	26 (30)
Antihypertensive therapy	78 (89)
Lipid lowering therapy	16 (18)
Antacid therapy	82 (94)

Categorical variables are indicated as number (percentage). Data are medians (interquartile range). ICH, intracerebral hemorrhage; IQR, interquartile range; LMWH, low molecular weight heparin; mRS, modified Rankin Scale; n, number; NIHSS, National Institutes of Health Stroke Scale.

## Thrombin generation in patients with ICH and in controls

**Figure 1:**
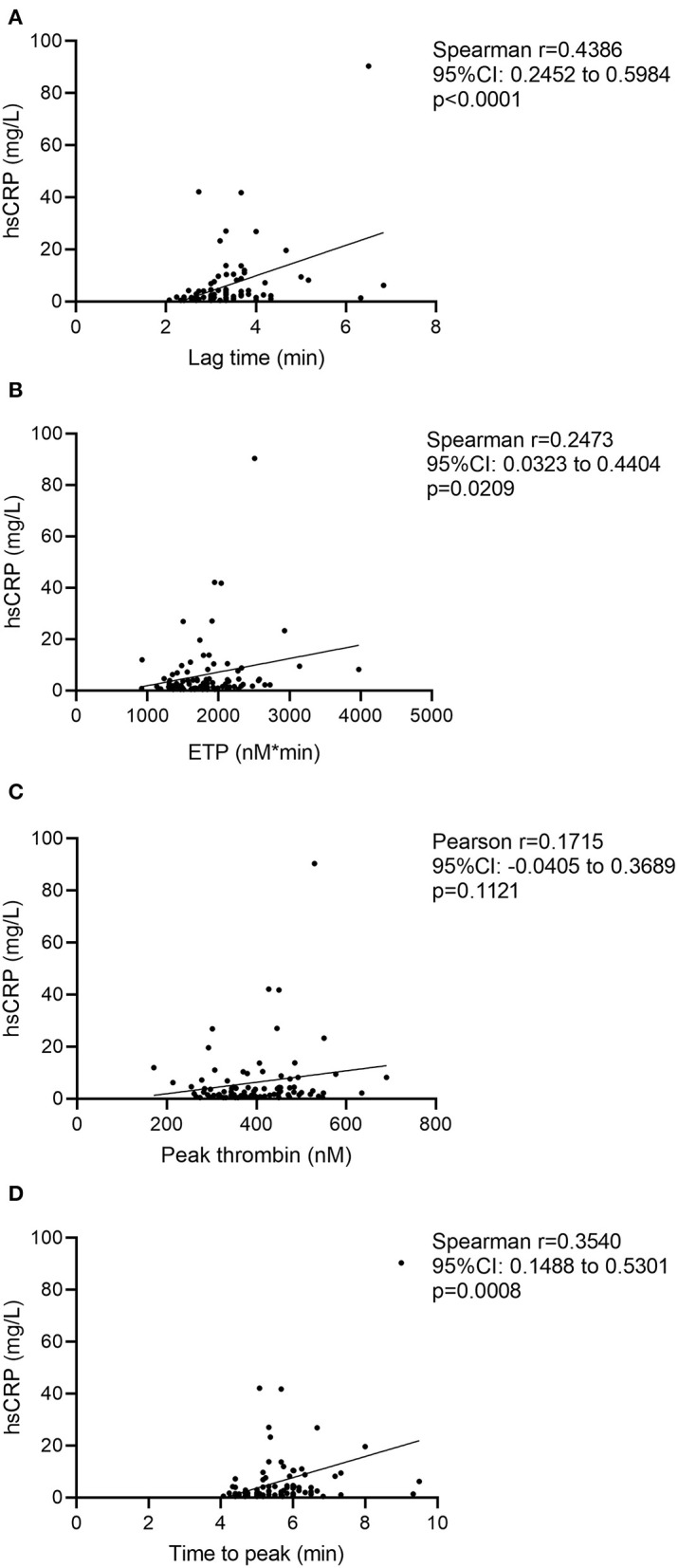
Correlation between TGA parameters and hsCRP levels in patients. Correlations between high-sensitivity C-reactive protein and lag time parameter **(A)**, endogenous thrombin potential **(B)**, peak thrombin **(C)**, and time to peak parameter **(D)** are shown. CI, confidence interval; ETP, endogenous thrombin potential; hsCRP: high-sensitivity C-reactive protein measurement.

The result of the TGA measurements in patients and in controls is shown in Table 3. Peak thrombin was significantly higher in patients as compared to controls (397.2 ± 93.9 nM vs. 306.3 ± 85.3 nM, respectively, *p* < 0.0001). Time to peak parameter was significantly shorter in patients with ICH [median: 5.5 (IQR: 5.0–6.0) min in patients vs. 6.5 (IQR: 5.6–7.8) min in controls, *p* < 0.0001]. Lag time and ETP did not differ between groups. Lag time, ETP, and time to peak parameters showed a significant positive correlation with CRP in patients (Figure 1) and in controls (Supplementary Tables 1, 2). Lag time, ETP, and time to peak parameters showed a similar significant positive correlation with fibrinogen in patients, but not in controls (Supplementary Tables 1, 2). In patients, the peak thrombin parameter showed a significant positive correlation with white blood cell count (r = 0.2385, 95% CI: 0.0293–0.4277, *p* = 0.0261) and a significant negative correlation with PT (r = −0.3080, 95% CI: −0.4871– −0.1041, *p* = 0.0037) and APTT (r = −0.4277, 95% CI:−0.5856– −0.2385, *p* < 0.0001) (Supplementary Table 1). D-dimer and FVIII activity did not show significant correlation with any of the TGA parameters in patients and in controls (Supplementary Tables 1, 2).

**Table 3:** Thrombin generation parameters in the studied cohort.

	**Patients** ***n* = 87**	**Controls** ***n* = 164**	***p***
Lag time (min)	3.3 (3.0–3.6)	3.3 (2.7–4.1)	0.4090
ETP (nM*min)	1,776 (1,491–2,099)	1,707 (1,472–1,973)	0.2374
Peak thrombin (nM)	397.2 ± 93.9	306.3 ± 85.3	<0.0001
Time to peak (min)	5.5 (5.0–6.0)	6.5 (5.6–7.8)	<0.0001

Data are mean ± SD or medians (interquartile ranges). ETP, endogenous thrombin potential; n, number.

**Figure 2:**
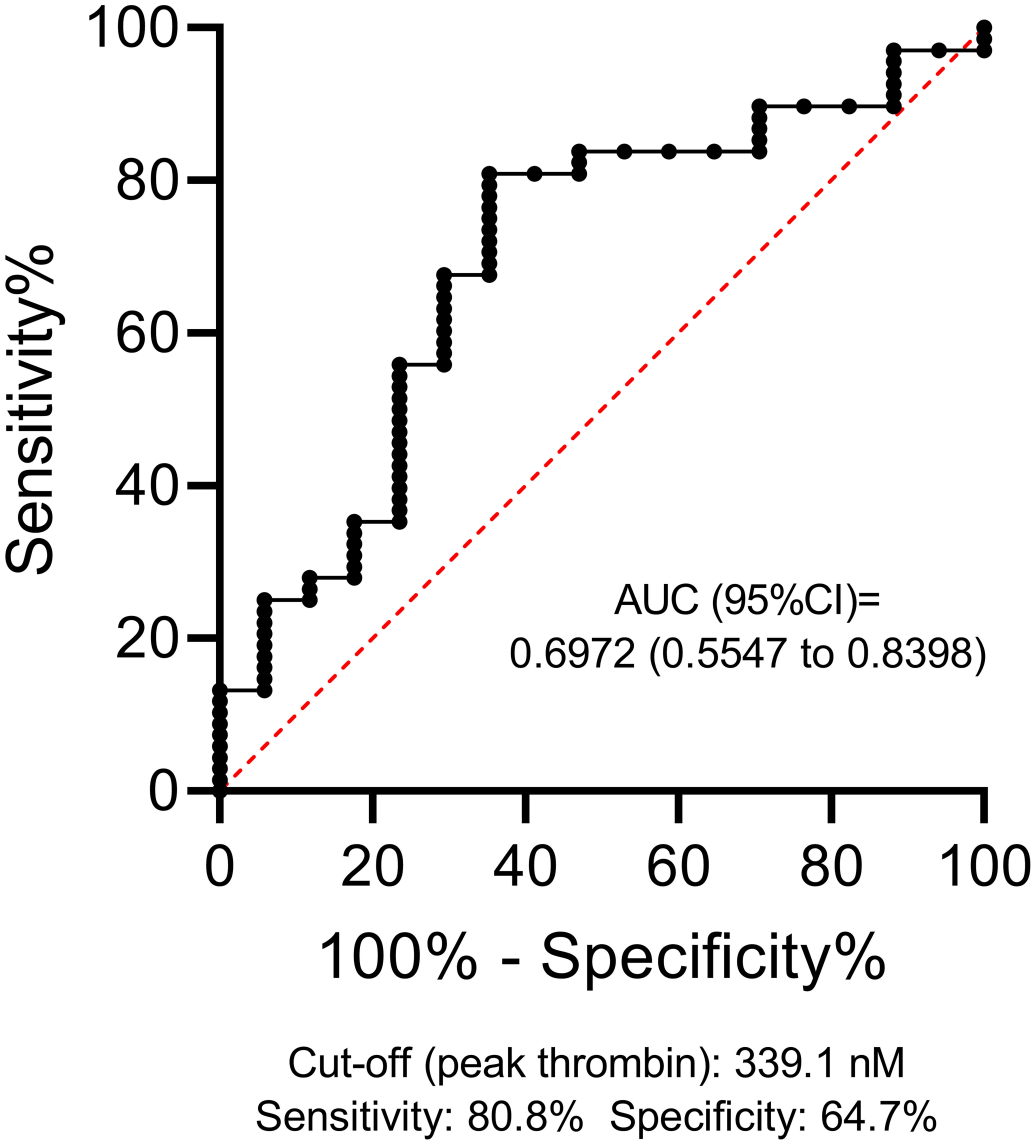
Receiver operator characteristics (ROC) curve of the peak thrombin parameter for estimating poor outcome (mRS 2–6) in patients with ICH. ROC curve and descriptive statistics, including best cut-off value as determined by the Youden's J statistics, are depicted for peak thrombin parameter. AUC, area under the curve; CI, confidence interval; ICH, intracerebral hemorrhage.

Stroke severity and short-term outcomes showed no association with TGA parameters (data not shown). Poor long-term outcome was significantly associated with a higher BMI, higher D-dimer levels, higher NIHSS on admission, larger estimated hemorrhage volume at any measured time-point, higher ICH score, higher peak thrombin parameter, and being a non-smoker (Table 4). In patients with worse long-term functional outcomes, peak thrombin was significantly higher as compared to those with favorable outcomes [mRS 2–6 median: 402.5 (IQR: 344.8–473.8) vs. mRS 0–1: 326.4 (294.2–416.1) nM, *p* = 0.0096] (Table 4). ROC analysis was performed to investigate the diagnostic performance and the optimal threshold of the peak thrombin parameter for predicting long-term outcomes (Figure 2). Based on the statistically optimal threshold of 339.1 nM peak thrombin, the sensitivity and specificity of peak thrombin parameter to determine mRS 2–6 as outcome were 80.8 and 64.7%, respectively. Using this threshold, poor outcome can be ruled out with a high negative value (NPV: 0.9016, 95% CI: 0.8016–0.9541; PPV: 0.4583, 95% CI: 0.2789–0.6493). In a binary logistic regression model including age, sex, BMI, smoking status, NIHSS on admission, D-dimer (>0.5 mg/L), and peak thrombin (>339.1 nM), only NIHSS and the peak thrombin parameters remained in the model as significant, independent predictors of poor outcome (OR: 1.458, 95% CI: 1.180–1.801, *p* < 0.001 per unit change in NIHSS and OR: 48.292, 95% CI: 4.667–499.720, *p* = 0.001, respectively). Lag time and time to peak showed a modest, significant negative correlation with intracerebral bleeding volume on admission (*r* = −0.2603, 95% CI: −0.4642– −0.0303, *p* = 0.0231 and *r* = −0.3698, 95% CI: −0.5542– −0.1509, *p* = 0.0010, respectively) (Table 5). During the follow-up of patients, hemorrhage volumes on day 90 showed significant positive association with the ETP and peak thrombin parameters (*r* = 0.3838, 95% CI: 0.0161–0.6600, *p* = 0.0363 and *r* = 0.5383, 95% CI: 0.2103–0.7574, *p* = 0.0021, respectively). FVIII activity showed no correlation with hemorrhage volumes (data not shown), whereas D-dimer levels correlated with day 14 estimated hemorrhage volumes only (*r* = 0.3504, 95% CI: 0.0860–0.5689, *p* = 0.0087). Mortality by days 14 and 90 was not associated with TG parameters, FVIII activity, or D-dimer levels in this cohort (data not shown). The peak thrombin parameter on admission was above the 339.1 nM threshold in all patients with VTE events during the follow-up (data not shown).

**Table 4:** Baseline clinical data and thrombin generation parameters according to long-term functional outcomes at 90 days post-event.

**Variables**	**mRS 0–1**	**mRS 2–6**	***p***
Number of individuals, *n*	17	69	-
Age, y, mean ± SD	62 ± 13	68 ± 12	0.0791
Male sex, *n* (%)	11 (65)	44 (64)	0.9480
Cerebrovascular risk factors, *n* (%)
Arterial hypertension	15 (88)	68 (99)	0.0983
Hyperlipidemia	13 (76)	37 (54)	0.1056
Diabetes mellitus	7 (41)	24 (35)	0.7787
Active smoker	8 (47)	8 (12)	0.0024
BMI, kg/m^2^, median (IQR)	24.04 (21.78–28–25)	27.31 (24.93–31.60)	0.0090
Laboratory parameters on admission, median (IQR) or mean ± SD
Serum glucose, mmol/L	7.4 (5.5–11.3)	7.2 (5.9–9.8)	0.9979
Triglyceride, mmol/L	1.5 (0.96–2.1)	1.5 (1.0–2.2)	0.9146
Total cholesterol, mmol/L	5.3 ± 1.2	4.8 ± 1.1	0.1422
HDL, mmol/L	1.5 (1.0–1.8)	1.4 (1.1–2.0)	0.4875
LDL, mmol/L	3.4 ± 1.1	2.9 ± 1.0	0.1040
Creatinine, μmol/L	69 (57–85)	68 (59–83)	0.8964
ASAT, U/L	20.0 (14.3–36.3)	23.5 (17.0–33.0)	0.3339
ALT, U/L	18.5 (13.0–27.8)	17.0 (14.0–23.5)	0.7296
LD, U/L	217 (191–245)	224 (199–258)	0.3714
hsCRP, mg/L	1.6 (0.9–3.9)	2.7 (1.2–7.9)	0.2173
WBC, G/L	6.9 (6.4–9.0)	8.6 (6.7–11.8)	0.1361
RBC, T/L	4.5 ± 0.4	4.7 ± 0.6	0.3347
Hemoglobin, g/L	144 ± 12	142 ± 17	0.6862
Platelet count, G/L	250 (158–274)	218 (182–269)	0.8840
PT, s	8.1 (7.8–8.2)	8.1 (7.8–8.6)	0.3723
INR	0.96 (0.93–0.98)	0.96 (0.93–1.02)	0.6031
APTT, s	27.5 (24.6–29.5)	26.5 (24.5–29.2)	0.5309
TT, s	16.6 (16.2–17.5)	16.6 (15.8–17.8)	0.9394
Fibrinogen, g/L	4.0 (3.2–4.4)	3.7 (3.0–4.5)	0.8810
D-dimer, mg/L	0.36 (0.28–0.58)	0.72 (0.48–1.3)	0.0013
FVIII activity, %	210 (152–232)	230 (155–294)	0.1158
Stroke severity on admission, NIHSS, median (IQR)	4 (4–6)	16 (11–21)	<0.0001
Estimated volume of hemorrhage, cm^3^, median (IQR)
On admission	2.3 (1.4–6.2)	17.0 (6.5–37.1)	<0.0001
Day 14	3.1 (1.4–4.8)	19.0 (3.9–29.5)	0.0008
Day 90	0 (0)	1.0 (0–6.5)	0.0120
ICH score, median (IQR)	0 (0–1)	2 (1–3)	0.0005
Thrombin generation parameters, median (IQR)
Lag time (min)	3.3 (2.8–3.8)	3.3 (3.0–3.67)	0.9978
ETP (nM*min)	1,666 (1,445–1,793)	1,832 (1,496–2,122)	0.1769
Peak thrombin (nM)	326.4 (294.2–416.1)	402.5 (344.8–473.8)	0.0096
Time to peak (min)	5.8 (5.0–6.4)	5.3 (5.0–6.0)	0.2177

Continuous variables are expressed as mean ± SD or median (interquartile range). Categorical variables are indicated as number (percentage). ALT, alanine aminotransferase; APTT, activated partial thromboplastin time; ASAT, aspartate aminotransferase; BMI, body mass index; ETP, endogenous thrombin potential; FVIII, coagulation factor VIII; HDL, high-density lipoprotein; hsCRP, high-sensitivity C-reactive protein measurement; ICH, intracerebral hemorrhage; INR, international normalized ratio; IQR, interquartile range; LD, lactate dehydrogenase; LDL, low-density lipoprotein; n, number; NIHSS, National Institutes of Health Stroke Scale; PT, prothrombin time; RBC, red blood cell count; SD, standard deviation; TT, thrombin time; WBC, white blood cell count.

**Table 5:** Correlation between estimated intracerebral hemorrhage volumes at admission and during follow-up and thrombin generation parameters at admission.

		**Estimated volume of hemorrhage**		
		**On admission**	**Day 14**	**Day 90**
Thrombin generation parameters	Lag time (min)	r = −0.2603 95% CI: −0.4642– −0.0303 *p* = **0.0231**	r = −0.2100 95% CI: −0.4566–0.0665 *p* = 0.1238	r = 0.0673 95% CI: −0.3104–0.4266 *p* = 0.7239
	ETP (nM*min)	r = −0.0771 95% CI: −0.3036–0.1575 *p* = 0.5076	r = −0.1175 95% CI: −0.3781–0.1604 *p* = 0.3930	r = 0.3838 95% CI: 0.0161–0.6600 *p* = **0.0363**
	Peak thrombin (nM)	r = 0.0723 95% CI: −0.1623–0.2992 *p* = 0.5346	r = 0.0170 95% CI: 0.2569–0.2885 *p* = 0.9016	r = 0.5383 95% CI: 0.2103–0.7574 *p* = **0.0021**
	Time to peak (min)	r= −0.3698 95% CI: −0.5542– −0.1509 *p* = **0.0010**	r= −0.2323 95% CI: −0.4749–0.0432 *p* = 0.0879	r= −0.0543 95% CI: −0.4159–0.3221 *p* = 0.7756

CI, confidence interval; ETP, endogenous thrombin potential. Bold numbers indicate values of statistical significance.

## Discussion

To our knowledge, this study shows for the first time that the TGA is associated with the evolution of hematoma in patients and it might be a promising tool to predict the outcome of ICH. Despite the clear benefit of diagnostic tests with acceptable predictive value regarding outcomes in patients with acute ICH stroke, surprisingly, few studies are available on this topic. Here, we show that the most relevant TGA parameters (e.g., peak thrombin and ETP) of patients on admission correlate with the estimated size of hematoma at 90 days post-event, which is an important parameter of outcome. According to our results, an elevated peak thrombin is associated with worse long-term functional outcome in patients, and this parameter proved to be a significant, independent predictor of long-term outcomes. Although one might expect a lower extent of thrombin generation in patients with ICH with hematoma growth and poor outcomes, these results are in line with previous studies and our current study revealing that an elevated D-dimer, indicating more extensive coagulation and breakdown of clots, is associated with poor outcomes in patients with ICH (6–8). Our study indicates that the TGA might be even more relevant in the prognosis of ICH as D-dimer, as in the backward logistic regression analysis including all relevant variables, only peak thrombin and NIHSS remained in the model as independent predictors of poor outcome (mRS 2–6). Our results clearly show a potential distortion of hemostatic balance in patients with ICH toward a hypercoagulable state. When comparing thrombin generation in patients and healthy controls, peak thrombin parameter was significantly higher in patients. Correlation between thrombin generation parameters and baseline laboratory characteristics revealed that the difference might be explained by the presence of higher inflammatory parameters in patients. As the major cause of ICH was assumed to be hypertension in all patients in this cohort, it can be surmised that a low-grade, chronic inflammation exerted by an enhanced atherosclerotic process might induce a higher extent of thrombin generation in this cohort. Although fibrinogen, an acute phase protein, was positively associated with most thrombin generation parameters in patients, fibrinogen levels were within the normal range in this cohort and showed no significant difference between groups of different outcomes, implying that fibrinogen itself might not be directly involved in this process. Interestingly, PT and APTT showed a moderately strong negative correlation with peak thrombin, implying that factors resulting in shortened PT and APTT might be associated with a higher peak of thrombin formation. Although FVIII was significantly higher in patients with ICH as compared to healthy controls, FVIII levels did not show a correlation with any TGA parameters. Further studies are required to reveal mechanisms contributing to the observed higher thrombin generation in patients with ICH.

Interestingly, the evolution of the intracerebral hematoma was associated with the extent of thrombin generation in this cohort. This information might serve as important knowledge in the future, as data on the factors that drive the enlargement or the dissolution of the bleeding are potentially important when designing future pharmacological therapies. As a first step, it is crucial to understand the underlying pathomechanism leading to poor outcomes in patients. Second, adequate diagnostic tools could serve as a prerequisite in such approaches.

Thrombin generation assay is a method that has been shown to be potentially useful to predict the outcomes in a wide range of pathologies where the hemostasis balance has been tilted (16–18). The assay has a list of benefits and limitations, and most importantly, optimal conditions of the test have not been clearly defined, as yet. An undisputable advantage of the assay is being a global test of clot formation, thus saving efforts to determine the levels of individual factors of hemostasis using laborious and time-consuming methods. TGA as a global test incorporates the multiple procoagulant and anticoagulant pathways in its final result, and therefore, it more likely represents the situation occurring in the *in vivo* environment. TGA is highly variable among individuals, and studies suggest that this may allow individualized treatment in patients with bleeding disorders or on anticoagulant therapy (21). TGA has been used to predict bleeding tendency in various clinical conditions. In hemophiliac patients and in hemophiliacs with inhibitors, its usefulness has been proved, and it has been a matter of debate whether the TGA might describe the risk of bleeding better than traditional tests (22–24). TGA, when performed preoperatively, was found to be useful in providing information predictive of blood loss after cardiac surgery (25). On the other hand, in patients with thrombotic disorders, the test is potentially useful to identify those at higher risk for recurrent thrombosis (21). Patients with stroke, particularly those with ICH, are at significant risk of venous thromboembolic events (VTEs) (21). In a recent prospective observational study, it has been shown that after adjusting for confounders, patients with hemorrhagic stroke have significantly higher risk of in-hospital VTE than patients with acute ischemic stroke (1). In this studied cohort, 4 VTE events were registered, despite the use of prophylactic anticoagulation in high-risk patients. As the exact underlying pathophysiology of the increased VTE risk in patients with ICH has not been clarified, our results might provide additional data to understand more about the hypercoagulable state in this patient population. Additional studies are warranted to find out whether the TGA or other hemostasis assays might provide help in the VTE risk assessment and the antithrombotic management of patients with ICH.

It must be noted that a major factor limiting the use of TGA in the clinical practice currently is the fact that despite the attempts to standardize the test, analytical challenges remain to be elaborated. In future studies, assay conditions might be further improved and standardized, allowing direct comparison between laboratories. The ultimate goal in the development and standardization of the TGA will be to generate standardized assay conditions that lead to highly sensitive and specific tests that aid clinical decision-making, while allowing interlaboratory comparison of larger datasets. Clinical studies testing the utility of various hemostasis tests in predicting the outcome of intracerebral hemorrhage are scarcely found in the literature as yet. Our study, similarly to a few previous studies testing hemostasis abnormalities in patient cohorts, is a hypothesis-generating study to obtain insights into whether TGA is associated with poor outcomes in patients with ICH (26–28). Further long-term follow-up studies are warranted to verify our results and optimize assay conditions.

## Conclusion

In patients with ICH, TG was increased as compared to healthy controls, which might be explained by the presence of higher inflammatory parameters in patients. Peak thrombin measured on admission might be a useful tool to predict outcomes in patients with ICH.

## Limitations

The results of this study should be interpreted in the context of its limitations and strengths. The sample size is limited; however, as compared to other published prospective studies of consecutive patients with non-traumatic, spontaneous ICH, involving the measurement of hemostasis biomarkers from admission samples, it is among the largest studies as yet. Nevertheless, the results presented here must be confirmed and validated by larger studies. The study was single-centered, which contributed to the limited sample size, but it had the advantages of uniform sample handling and patient care, and the major benefit that only one patient was lost to follow-up. As the TGA has no reference ranges provided by the distributor as yet, TGA results of healthy controls were included in this study. Despite our best efforts, based on the criteria of healthy control group, the age and sex of patients and controls were significantly different, which limits the direct comparison of results between groups. Assessment of TGA with different reagents (e.g., with low tissue factor concentration) was out of the scope of this study. The study was not designed to find out whether the TGA or other hemostasis assays might provide help in the assessment of VTE risk and the personalized antithrombotic management of patients with ICH; therefore, additional studies are warranted to focus on this aspect.

## Data availability statement

The raw data supporting the conclusions of this article will be made available by the authors, without undue reservation.

## Ethics statement

The studies involving human participants were reviewed and approved by the Institutional Ethics Committee of the University of Debrecen and the Ethics Committee of the National Medical Research Council. The patients/participants provided their written informed consent to participate in this study.

## Author contributions

LL and RO-K collected clinical samples, performed experiments, analyzed, and interpreted the data. TÁ, IF, KF, MH, and JT collected clinical data and analyzed and interpreted the data. LC designed the research and interpreted the data. ZB designed the research, analyzed and interpreted the data, and wrote the manuscript. All authors have read and approved the final manuscript.

## Funding

This work was supported by grants from the National Research, Development and Innovation Fund (K120042 and FK128582), by GINOP-2.3.2-15-2016-00043, the Lóránd Eötvös Research Network (ELKH-DE Cerebrovascular Research Group), and by ÚNKP 20-3-I-DE-220.

## Conflict of interest

The authors declare that the research was conducted in the absence of any commercial or financial relationships that could be construed as a potential conflict of interest.

## Publisher's note

All claims expressed in this article are solely those of the authors and do not necessarily represent those of their affiliated organizations, or those of the publisher, the editors and the reviewers. Any product that may be evaluated in this article, or claim that may be made by its manufacturer, is not guaranteed or endorsed by the publisher.
