# Supplementary material for: Thrombin generation as a predictor of outcomes in patients with non-traumatic intracerebral hemorrhage
Source: Front Neurol. 2022 Aug 18;13:912664. doi: 10.3389/fneur.2022.912664 (PMC9436391; doi:10.3389/fneur.2022.912664)
Supplement: Supplementary file 1 [file Data_Sheet_1.docx]

**Supplementary Table 1. Correlations between baseline antropometric data, laboratory parameters and thrombin generation parameters in patients**

|  | | **Age (y)** | **BMI (kg/m^2^)** | **hsCRP (mg/L)** | **WBC (G/L)** | **Platelet count (G/L)** |
| --- | --- | --- | --- | --- | --- | --- |
| Thrombin generation parameters | Lag time (min) | r= 0.1277  95% CI:  -0.0915 to 0.3351  p=0.2385 | r= 0.1921  95% CI:  -0.0339 to 0.3995  p=0.0858 | r= 0.4386  95% CI:  0.2452 to 0.5984  p<**0.0001** | r= 0.1155  95% CI:  -0.1038 to 0.3241  p=0.2866 | r= 0.0873  95% CI:  -0.1318 to 0.2984  p=0.4210 |
|  | ETP (nM*min) | r= -0.0166  95% CI:  -0.2265 to 0.1947  p=0.8784 | r= -0.0227  95% CI:  -0.2460 to 0.2029  p=0.8405 | r= 0.2473  95% CI:  0.0323 to 0.4404  p=**0.0209** | r= 0.0847  95% CI:  -0.1344 to 0.2960  p=0.4349 | r= 0.1154  95% CI:  -0.1039 to 0.3239  p=0.2873 |
|  | Peak thrombin (nM) | r= -0.0142  95% CI:  -0.2242 to 0.1970  p=0.8958 | r= -0.0316  95% CI:  -0.2483 to 0.1880  p=0.7792 | r= 0.1715  95% CI:  -0.0405 to 0.3689  p=0.1121 | r= 0.2385  95% CI:  0.0293 to 0.4277  p=**0.0261** | r= 0.0042  95% CI:  -0.2066 to 0.2147  p=0.9689 |
|  | Time to peak (min) | r= 0.0863  95% CI:  -0.1328 to 0.2975  p=0.4263 | r= 0.0710  95% CI:  -0.1560 to 0.2910  p=0.5285 | r= 0.3540  95% CI:  0.1488 to 0.5301  p=**0.0008** | r= 0.0420  95% CI:  -0.1762 to 0.2564  p=0.6988 | r= 0.1811  95% CI:  -0.0370 to 0.3828  p=0.0932 |

**Supplementary Table 1. Continued**

|  | | **PT (s)** | **APTT (s)** | **TT (s)** | **Fibrinogen (g/L)** | **D-dimer (mg/L)** | **FVIII (%)** |
| --- | --- | --- | --- | --- | --- | --- | --- |
| Thrombin generation parameters | Lag time (min) | r= -0.0512  95% CI:  -0.2650 to 0.1673  p=0.6375 | r= 0.3476  95% CI:  0.1416 to 0.5248  p=**0.0010** | r= 0.1067  95% CI:  -0.1125 to 0.3161  p=0.3251 | r= 0.4848  95% CI:  0.2933 to 0.6388  p<**0.0001** | r= -0.0190  95% CI:  -0.2360 to 0.1997  p=0.8619 | r= -0.1011  95% CI:  -0.3185 to 0.1263  p=0.3690 |
|  | ETP (nM*min) | r= -0.1405  95% CI:  -0.3466 to 0.0786  p=0.1944 | r= -0.1560  95% CI:  -0.3605 to 0.0627  p=0.1490 | r= -0.1629  95% CI:  -0.3667 to 0.0557  p=0.1316 | r= 0.3520  95% CI:  0.1398 to 0.5333  p=**0.0012** | r= 0.0216  95% CI:  -0.1973 to 0.2384  p=0.8435 | r= -0.1266  95% CI:  -0.3415 to 0.1008  p=0.2600 |
|  | Peak thrombin (nM) | r= -0.3080  95% CI:  -0.4871 to -0.1041  p=**0.0037** | r= -0.4277  95% CI:  -0.5856 to -0.2385  p<**0.0001** | r= -0.2039  95% CI:  -0.3974 to 0.0071  p=0.0582 | r= 0.1728  95% CI:  -0.0459 to 0.3757  p=0.1205 | r= -0.0596  95% CI:  -0.2681 to 0.1542  p=0.5853 | r= -0.0613  95% CI:  -0.2760 to 0.1591  p=0.5862 |
|  | Time to peak (min) | r= -0.2219  95% CI:  -0.4185 to -0-0055  p=**0.0388** | r= 0.3806  95% CI:  0.1787 to 0.5518  p=**0.0003** | r= 0.0528  95% CI:  -0.1657 to 0.2665  p=0.6266 | r= 0.4890  95% CI:  0.2983 to 0.6421  p<**0.0001** | r= -0.0166  95% CI:  -0.2337 to 0.2021  p=0.8795 | r= -0.1296  95% CI:  -0.3441 to 0.0978  p=0.2490 |

Spearman or Pearson correlation. APTT, activated partial thromboplastin time; BMI, body mass index; CI, confidence interval; ETP, endogenous thrombin potential; FVIII, coagulation factor VIII; hsCRP, high sensitivity C-reactive protein measurement; PT, prothrombin time; TT, thrombin time; WBC, white blood cell count.

**Supplementary Table 2. Correlations between baseline antropometric data, laboratory parameters and thrombin generation parameters in healthy controls**

|  | | **Age (y)** | **BMI (kg/m^2^)** | **hsCRP (mg/L)** | **WBC (G/L)** | **Platelet count (G/L)** |
| --- | --- | --- | --- | --- | --- | --- |
| Thrombin generation parameters | Lag time (min) | r= 0.1771  95% CI:  0.0136 to 0.3313  p=**0.0291** | r= -0.3771  95% CI:  -0.5102 to -0.2262  p<**0.0001** | r= 0.2511  95% CI:  0.0905 to 0.3990  p=**0.0019** | r= 0.0893  95%CI:  -0.1285 to 0.2991  p=0.4077 | r= 0.1241  95% CI:  -0.0938 to 0.3307  p=0.2493 |
|  | ETP (nM*min) | r= 0.0144  95%CI:  -0.1497 to 0.1779  p=0.8595 | r= -0.3283  95%CI:  -0.4679 to -0.1728  p<**0.0001** | r= 0.3610  95%CI:  0.2091 to 0.4960  p**<0.0001** | r= 0.0819  95%CI:  -0.1359 to 0.2922  p=0.4480 | r= 0.0385  95% CI:  -0.1784 to 0.2519  p=0.7212 |
|  | Peak thrombin (nM) | r= -0.0865  95%CI:  -0.2424 to 0.0736  p=0.2890 | r= -0.0224  95%CI:  -0.1821 to 0.1383  p=0.7847 | r= 0.0355  95%CI:  -0.1249 to 0.1942  p=0.6644 | r= -0.0251  95%CI:  -0.2333 to 0.1853  p=0.8162 | r= -0.0183  95% CI:  -0.2269 to 0.1919  p=0.8655 |
|  | Time to peak (min) | r= 0.1872  95%CI:  0.0241 to 0.3406  p=**0.0209** | r= -0.3393  95%CI:  -0.4775 to -0.1847  p<**0.0001** | r= 0.1931  95%CI:  0.0296 to 0.3464  p=**0.0175** | r= 0.0876  95%CI:  -0.1303 to 0.2974  p=0.4170 | r= 0.1040  95% CI:  -0.1140 to 0.3124  p=0.3350 |

**Supplementary Table 2. Continued**

|  | | **PT (s)** | **APTT (s)** | **TT (s)** | **Fibrinogen (g/L)** | **D-dimer (mg/L)** | **FVIII (%)** |
| --- | --- | --- | --- | --- | --- | --- | --- |
| Thrombin generation parameters | Lag time (min) | r= 0.2529  95% CI:  0.0686 to 0.4206  p=**0.0062** | r= -0.1733  95%CI:  -0.3998 to 0.0732  p=0.1545 | r= -0.0234  95% CI:  -0.2101 to 0.1648  p=0.8024 | r= 0.0551  95% CI:  -0.1908 to 0.2946  p=0.6526 | r= 0.0787  95%CI:  -0.1130 to 0.2649  p=0.4069 | r= 0.0977  95% CI:  -0.0940 to 0.2825  p=0.3031 |
|  | ETP (nM*min) | r= 0.2270  95% CI:  0.0412 to 0.3977  p=**0.0143** | r= 0.0052  95%CI:  -0.2384 to 0.2484  p=0.9657 | r= -0.0373  95% CI:  -0.2233 to 0.1513  p=0.6907 | r= 0.2138  95% CI:  -0.0312 to 0.4345  p=0.0778 | r= -0.1465  95%CI:  -0.3275 to 0.0447  p=0.1214 | r= -0.0105  95% CI:  -0.2002 to 0.1799  p=0.9122 |
|  | Peak thrombin (nM) | r= -0.0148  95% CI:  -0.1966 to 0.1679  p=0.8743 | r= 0.0964  95%CI:  -0.1435 to 0.3257  p=0.4306 | r= -0.0559  95% CI:  -0.2359 to 0.1277  p=0.5509 | r= 0.3631  95% CI:  0.1383 to 0.5523  p=**0.0022** | r= -0.1408  95%CI:  -0.3173 to 0.0450  p=0.1367 | r= -0.0610  95% CI:  -0.2430 to 0.1251  p=0.5210 |
|  | Time to peak (min) | r= 0.2269  95% CI:  0.0410 to 0.3976  p=**0.0143** | r= -0.1841  95%CI:  -0.4092 to 0.0620  p=0.1299 | r= -0.0228  95% CI:  -0.2096 to 0.1654  p=0.8073 | r= -0.0620  95% CI:  -0.3009 to 0.1841  p=0.6124 | r= 0.1089  95%CI:  -0.0828 to 0.2929  p=0.2507 | r= 0.1203  95% CI:  -0.0713 to 0.3034  p=0.2043 |

Spearman or Pearson correlation. APTT, activated partial thromboplastin time; BMI, body mass index; CI, confidence interval; ETP, endogenous thrombin potential; FVIII, coagulation factor VIII; hsCRP, high sensitivity C-reactive protein measurement; PT, prothrombin time; TT, thrombin time; WBC, white blood cell count
